# Targeting myocardial equilibrative nucleoside transporter ENT1 provides cardioprotection by enhancing myeloid Adora2b signaling

**DOI:** 10.1172/jci.insight.166011

**Published:** 2023-06-08

**Authors:** Wei Ruan, Jiwen Li, Seungwon Choi, Xinxin Ma, Yafen Liang, Ragini Nair, Xiaoyi Yuan, Tingting W. Mills, Holger K. Eltzschig

**Affiliations:** 1 Department of Anesthesiology, Critical Care and Pain Medicine, The University of Texas Health Science Center at Houston, McGovern Medical School, Houston, Texas, USA.; 2 Department of Anesthesiology, Second Xiangya Hospital, Central South University, Changsha, China.; 3 Department of Cardiac Surgery, Sir Run Run Shaw Hospital, School of Medicine, Zhejiang University, Hangzhou, China.; 4 Department of Biochemistry and Molecular Biology, The University of Texas Health Science Center at Houston, Houston, Texas, USA.

**Keywords:** Cardiology, Inflammation, Cardiovascular disease, Hypoxia

## Abstract

Previous studies implicate extracellular adenosine signaling in attenuating myocardial ischemia and reperfusion injury (IRI). This extracellular adenosine signaling is terminated by its uptake into cells by equilibrative nucleoside transporters (ENTs). Thus, we hypothesized that targeting ENTs would function to increase cardiac adenosine signaling and concomitant cardioprotection against IRI. Mice were exposed to myocardial ischemia and reperfusion injury. Myocardial injury was attenuated in mice treated with the nonspecific ENT inhibitor dipyridamole. A comparison of mice with global *Ent1* or *Ent2* deletion showed cardioprotection only in *Ent1^–/–^* mice. Moreover, studies with tissue-specific *Ent* deletion revealed that mice with myocyte-specific *Ent1* deletion (*Ent1^loxP/loxP^* Myosin Cre^+^ mice) experienced smaller infarct sizes. Measurements of cardiac adenosine levels demonstrated that postischemic elevations of adenosine persisted during reperfusion after targeting ENTs. Finally, studies in mice with global or myeloid-specific deletion of the *Adora2b* adenosine receptor (*Adora2b^loxP/loxP^* LysM Cre^+^ mice) implied that Adora2b signaling on myeloid-inflammatory cells in cardioprotection provided by ENT inhibition. These studies reveal a previously unrecognized role for myocyte-specific ENT1 in cardioprotection by enhancing myeloid-dependent Adora2b signaling during reperfusion. Extension of these findings implicates adenosine transporter inhibitors in cardioprotection against ischemia and reperfusion injury.

## Graphical abstract

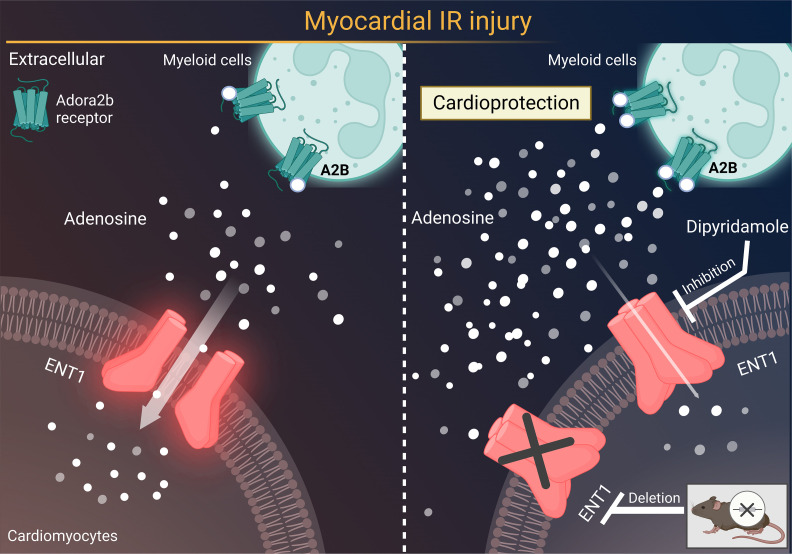

## Introduction

Myocardial ischemia and reperfusion injury (IRI) is among the leading causes of cardiovascular morbidity and mortality worldwide (1, 2). This pathologic condition is characterized by an obstruction of blood flow to the heart, followed by subsequent restoration of blood supply (3). Typically, this occlusion of coronary blood flow is caused by embolic occlusion of the vessel, which in turn creates an imbalance in the metabolic supply/demand ratio and results in profound myocardial hypoxia. When blood flow is restored spontaneously or due to coronary intervention, reoxygenation is associated with severe myocardial inflammation, referred to as myocardial reperfusion injury (3).

Previous research implicated the extracellular adenosine signaling in providing cardioprotection from IRI (4, 5) and promoting cardiac wound healing (6). Extracellular adenosine generation is increased during myocardial ischemia, where tissue hypoxia promotes adenosine generation and signaling (7, 8). Extracellular adenosine can function by binding to its receptors, including the Adora1, Adora2a, Adora2b, and Adora3 adenosine receptors (9, 10). When given i.v., the Adora1 adenosine receptor has been implicated in mediating the heart rate–slowing effects of adenosine treatment of supraventricular tachycardia (11). Particularly the Adora2a and Adoar2b receptors have been shown to dampen inflammatory responses (9, 12) and promote tissue protection from IRI (13–15). Adenosine signaling is terminated by adenosine uptake from the outside of the cells toward the inside through so-called equilibrative nucleoside transporters (ENTs) (16). Since adenosine is elevated during myocardial ischemia, the gradient across the cell membrane favors adenosine flow from the outside toward the inside (16). Conversely, pharmacologic blockade or genetic deletion of ENTs usually results in elevated extracellular adenosine levels and enhanced adenosine signaling (17–19). Previous studies have implicated the ENT inhibitor dipyridamole in cardioprotection, for example, by potentiating the infarct-size limiting effects of ischemic preconditioning (20, 21). However, the specific molecular pathways that facilitate ENT inhibition–mediated cardioprotection are elusive. In the present studies, we used genetic approaches in murine models of global and tissue-specific deletion of ENTs to address the functional roles of ENTs during myocardial IRI.

## Results

### Treatment with ENT inhibitor dipyridamole attenuates myocardial injury.

**Figure 1:**
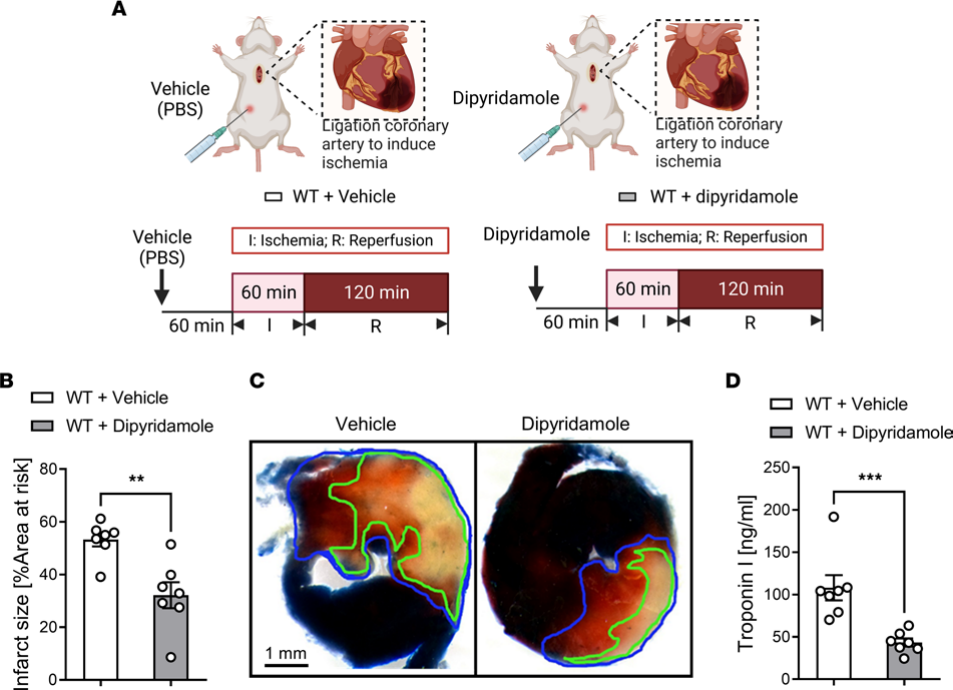
Treatment with ENT inhibitor dipyridamole provides cardioprotection. (**A**) Experimental strategy. C57BL/6J mice were given either PBS (WT + vehicle group) or dipyridamole (5 mg/kg, i.p.) (WT + dipyridamole group) 1 hour prior to in situ myocardial ischemia (60 minutes) and reperfusion (2 hours). (**B**) Infarct sizes in vehicle- or dipyridamole-treated mice (*n* = 7; 2-tailed unpaired *t* test, **P < 0.01). (**C**) Representative images after Evans blue injection and subsequent TTC staining. The infarct area is outlined by a green line; AAR is outlined by a blue line. Scale bar: 1 mm. (**D**) Troponin I (cTnI) levels (*n* = 7; Mann-Whitney *U* test, ***P < 0.001). Data are shown as mean ± SEM. Each dot represents 1 mouse.

Previous studies have indicated that inhibiting adenosine transporters could function to enhance the signaling effects of extracellular adenosine (16, 17). Extracellular adenosine signaling has been implicated in cardioprotection against IRI by activating adenosine receptors (22–24). Therefore, we pursued studies targeting adenosine transporters as a means of enhancing adenosine-dependent cardioprotection during IRI. The 2 predominant adenosine transporters implicated in terminating extracellular adenosine signaling during hypoxia or ischemia are ENT1 (25, 26) and ENT2 (18, 26, 27). Therefore, we pursued pharmacologic studies with dipyridamole, an effective adenosine transport inhibitor targeting both ENT1 and ENT2. Based on previous studies from our laboratory (17, 18), mice were pretreated with dipyridamole, 5 mg/kg, i.p., or corresponding vehicle control (PBS) 60 minutes prior to the onset of myocardial ischemia. Subsequently, we exposed mice to 60 minutes of in situ myocardial ischemia, followed by 2 hours of reperfusion, as previously described (22, 28, 29). After 2 hours of reperfusion, we assessed myocardial injury by estimating infarct sizes as a percentage of the area at risk (AAR) using the 2,3,5-Triphenyltetrazolium chloride (TTC) staining approach and measured the serum troponin I level as a myocardial injury marker (Figure 1A). Mice treated with dipyridamole experienced significantly smaller infarct sizes than mice receiving vehicle control (Figure 1, B and C). Similarly, the troponin I level was lower in dipyridamole-treated mice (Figure 1D). Taken together, these studies indicate that treatment with the nonspecific ENT inhibitor dipyridamole is associated with cardioprotection against myocardial IRI.

### Global deletion of Ent1 confers ENT-dependent cardioprotection.

**Figure 2:**
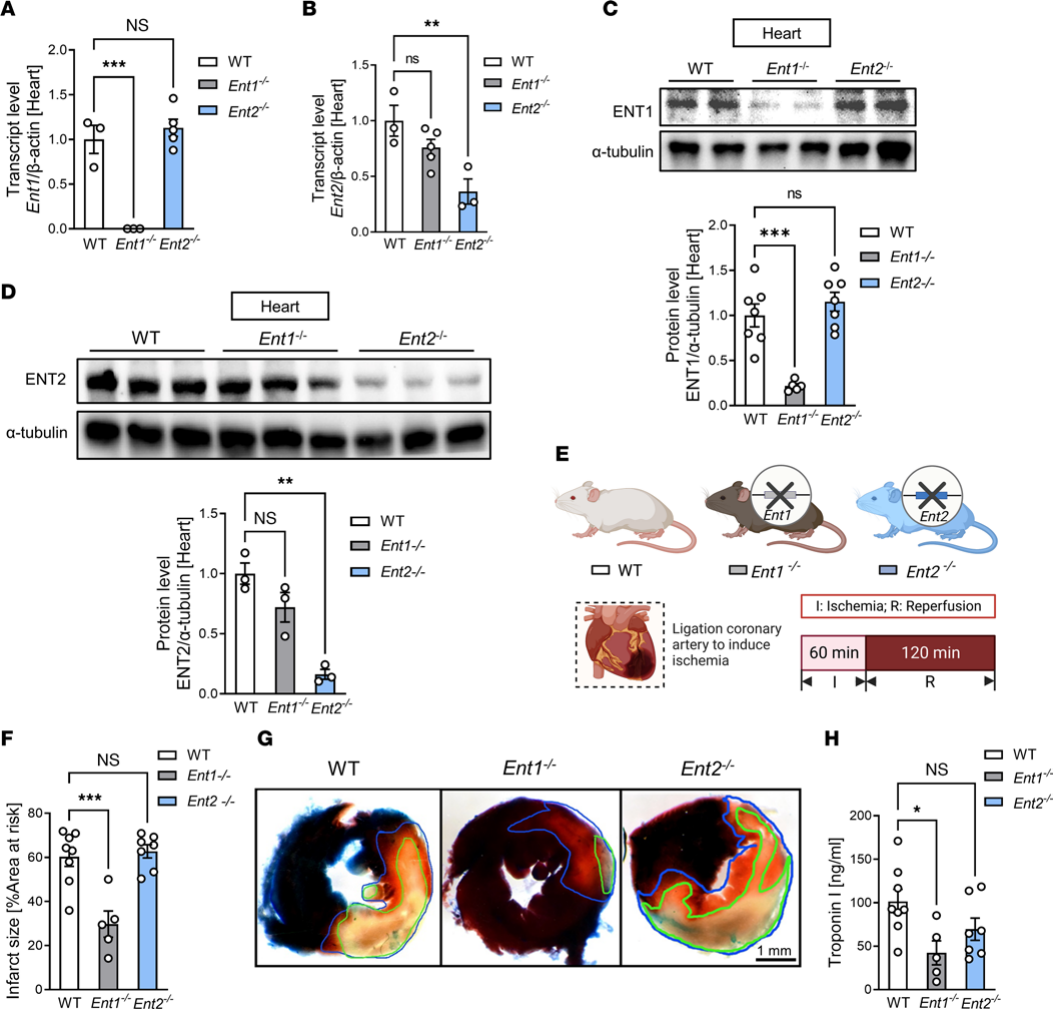
Global deletion of *Ent1*, but not *Ent2*, confers ENT-dependent cardioprotection. (**A** and **B**) *Ent1* (**A**) or *Ent2* (**B**) transcript levels in the heart of WT, *Ent1^–/–^*, or *Ent2^–/–^* mice (*n* = 3–5; 1-way ANOVA, **P < 0.01, ***P < 0.001 in Bonferroni’s multiple-comparison test). (**C**) Cardiac ENT1 protein by Western blot analysis. (*n* = 5–7; 1-way ANOVA, ***P < 0.001 in Bonferroni’s multiple-comparison test). (**D**) Cardiac ENT2 protein by Western blot analysis (*n* = 3; 1-way ANOVA, **P < 0.01 in Bonferroni’s multiple-comparison test). (**E**) Experimental strategy for the murine myocardial IRI in WT, *Ent1^–/–^*, or *Ent2^–/–^* mice. (**F**) Myocardial infarct sizes in WT, *Ent1^–/–^*, or *Ent2^–/–^* mice (*n* = 8 for WT, *n* = 5 for *Ent1^–/–^*, *n* = 7 for *Ent2^–/–^*, 1-way ANOVA, ***P < 0.001 in Bonferroni’s multiple-comparison test). (**G**) Representative images of left ventricles stained by Evans blue and TTC. The infarct area is outlined by a green line; AAR is outlined by a blue line. Scale bar: 1 mm. (**H**) cTnI levels after myocardial injury (*n* = 8 for WT, *n* = 5 for *Ent1^–/–^*, *n* = 7 for *Ent2^–/–^*, 1-way ANOVA, *P < 0.05 in Bonferroni’s multiple-comparison test). Data are shown as mean ± SEM. Each dot represents 1 mouse.

ENTs are widely distributed throughout diverse tissue types, exhibiting variable degrees of protein or mRNA expression levels in specific cell populations. According to the Human Protein Atlas, hENT1 protein and mRNA are considerably enriched in myocytes, fibroblasts, endothelial cells, and smooth muscle cells in the heart, whereas hENT2 displays relatively lower levels of abundance in myocytes of the heart (http://www.proteinatlas.org; ref 30). Here, we first utilized previously characterized *Ent1*–global KO mice (*Ent1^–/–^* mice) (17, 31) and *Ent2*–global KO mice (*Ent2^–/–^* mice) to investigate the respective contributions of different ENTs in mediating cardioprotective effects. Measurement of *Ent1* transcript level in the mouse heart revealed essentially undetectable *Ent1* mRNA levels in *Ent1^–/–^* mice (Figure 2A), while *Ent2* transcript level remained intact (Figure 2B). In addition, we generated *Ent2^–/–^* mice by crossing previously described mice with a “floxed” *Ent2* allele (17, 18) with germline Cre^+^ mice. As anticipated, *Ent2^–/–^* mice showed diminished expression of *Ent2* in the heart (Figure 2B). Subsequent Western blot experiments confirmed similar alterations in the specific knockdown effects for the protein levels of ENT1 or ENT2 (Figure 2, C and D). Next, we exposed *Ent1^–/–^*, *Ent2^–/–^*, or WT control animals to myocardial IRI, and we found attenuated infarct sizes in *Ent1^–/–^* mice while *Ent2^–/–^* mice experienced similar injury levels as WT controls (Figure 2, E–G). Similarly, serum troponin I levels were attenuated in *Ent1^–/–^* mice compared with *Ent2^–/–^* mice or WT controls (Figure 2H). Together, the above studies highlight a selective role of ENT1 for cardioprotection against IRI.

### Tissue-specific deletion of Ent1 identifies cardiac myocytes as an important source for Ent1-dependent cardioprotection.

**Figure 3:**
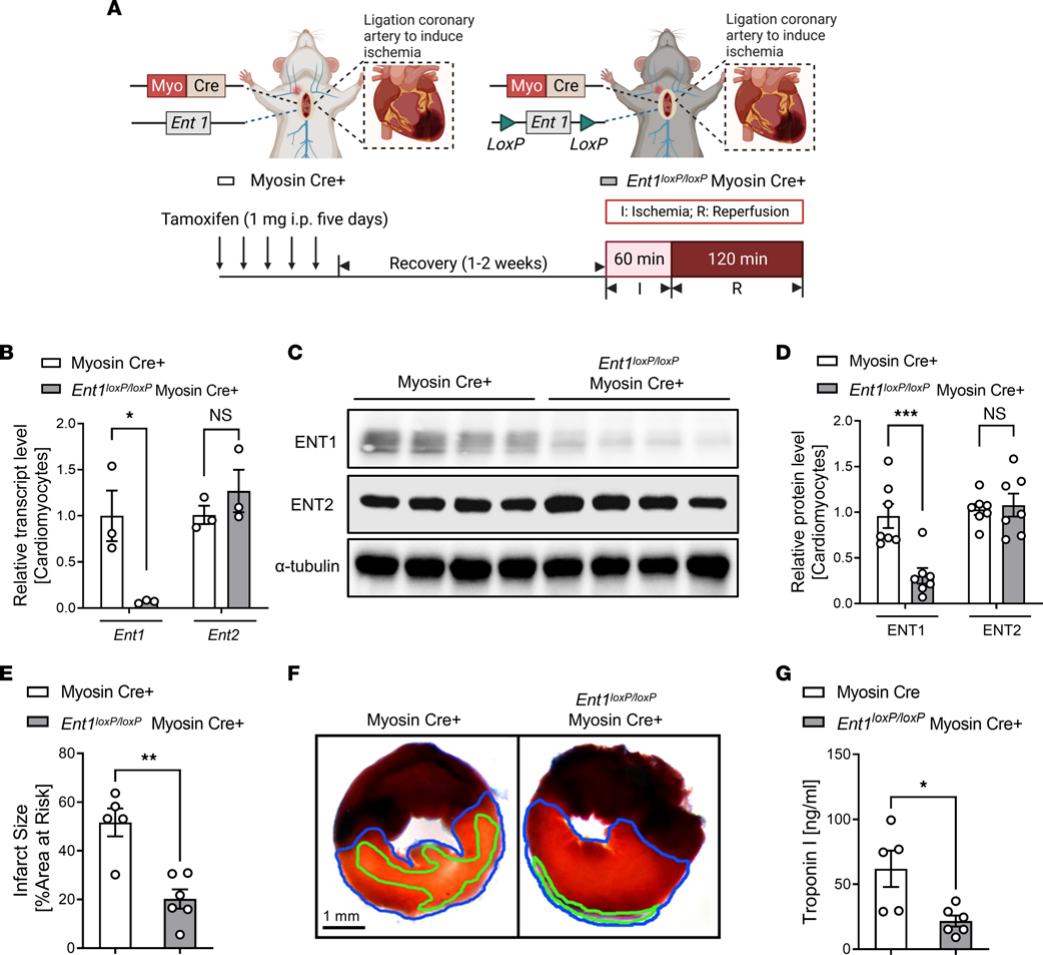
Tissue-specific deletion of *Ent1* identifies cardiac myocytes as an important source for *Ent1*-dependent cardioprotection. (**A**) Experimental approach to study myocardial ischemia (60 minutes) and reperfusion (2 hours) injury in control (Myosin Cre^+^) or *Ent1^loxP/loxP^* Myosin Cre^+^ mice. (**B**) *Ent1* or *Ent2* transcript levels in isolated cardiomyocytes in Myosin Cre^+^ or *Ent1^loxP/loxP^* Myosin Cre^+^ mice (*n* = 3; 2-way ANOVA, *P < 0.05 in Bonferroni’s multiple-comparison test). (**C**) ENT1 or ENT2 protein levels by Western blot analysis. (**D**) Quantification of **C** (*n* = 7; 2-way ANOVA, ***P < 0.001 in Bonferroni’s multiple-comparison test). (**E**) Infarct sizes in Myosin Cre^+^ or *Ent1^loxP/loxP^* Myosin Cre^+^ mice (*n* = 5 for Myosin Cre^+^, *n* = 6 for *Ent1^loxP/loxP^* Myosin Cre^+^, 2-tailed unpaired *t* test, **P < 0.01). (**F**) Representative TTC staining. The infarct area is outlined by a green line; AAR is outlined by a blue line. Scale bar: 1 mm. (**G**) cTnI levels (*n* = 5 for Myosin Cre^+^, *n* = 6 for *Ent1^loxP/loxP^* Myosin Cre^+^, Welch’s *t* test, *P < 0.05). Data are shown as mean ± SEM. Each dot represents 1 mouse.

**Figure 4:**
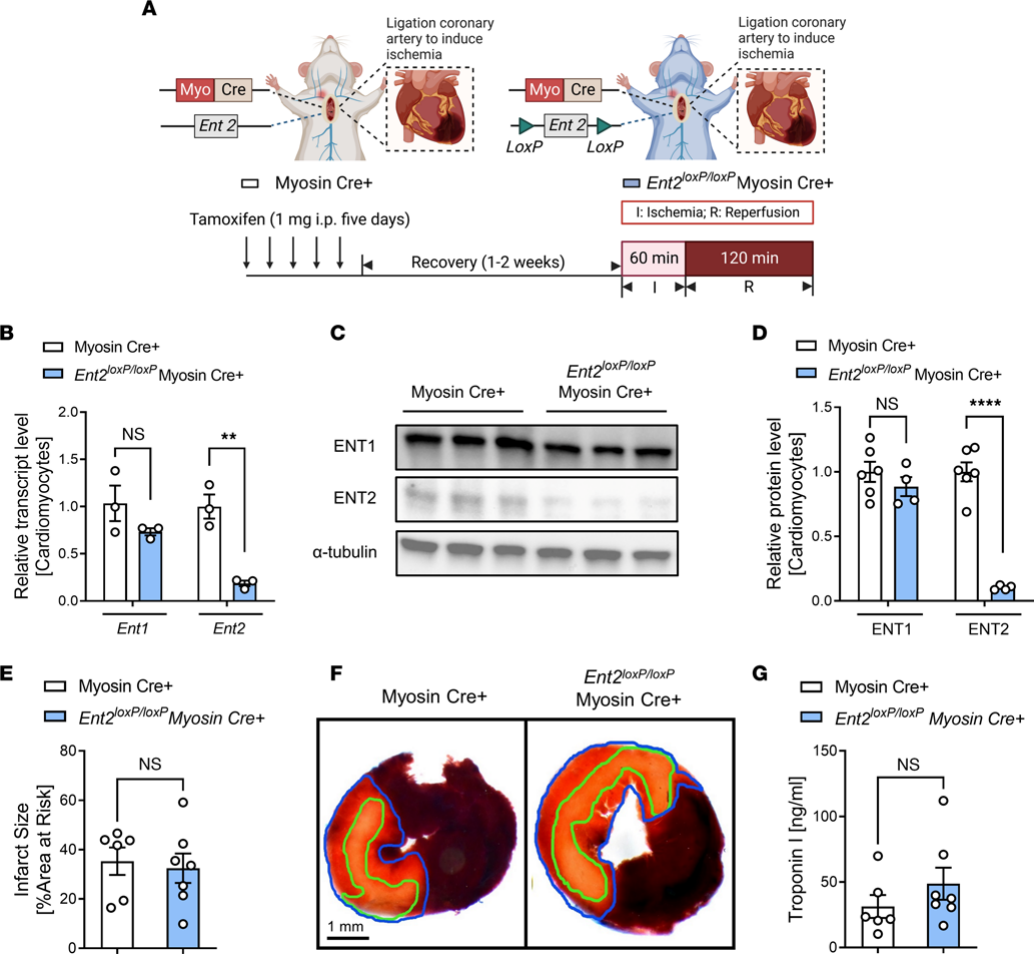
Tissue-specific deletion of *Ent2* is not associated with cardioprotection. (**A**) Experimental set-up to study myocardial ischemia (60 minutes) and reperfusion (2 hours) injury in control (Myosin Cre^+^) or *Ent2^loxP/loxP^* Myosin Cre^+^ mice. (**B**) *Ent1* or *Ent2* transcript levels were measured from isolated cardiomyocytes from Myosin Cre^+^ or *Ent2^loxP/loxP^* Myosin Cre^+^ mice (*n* = 3; 2-way ANOVA, **P < 0.01 in Bonferroni’s multiple-comparison test). (**C**) ENT1 or ENT2 protein levels by Western blot analysis. (**D**) Quantification of **C** (*n* = 4–6; 2-way ANOVA, ***P < 0.001 in Bonferroni’s multiple-comparison test). (**E**) Infarct sizes in Myosin Cre^+^ or *Ent2^loxP/loxP^* Myosin Cre^+^ mice (*n* = 6 for Myosin Cre^+^, *n* = 7 for *Ent2^loxP/loxP^* Myosin Cre^+^, Mann-Whitney *U* test). (**F**) Representative images of infarct staining. The infarct area is outlined by a green line; AAR is outlined by a blue line. Scale bar: 1 mm. (**G**) cTnI levels after surgery (*n* = 6 for Myosin Cre^+^, *n* = 7 for *Ent1^loxP/loxP^* Myosin Cre^+^, 2-tailed unpaired *t* test). Data are shown as mean ± SEM. Each dot represents 1 mouse.

We next conducted studies to identify critical tissue compartments responsible for the observed protection mediated by *Ent1* deletion. Given the predominant expression of ENT1 in cardiomyocytes in the heart (30), we first pursued functional studies in mice with myocyte-specific *Ent1* deletion by crossing mice with a “floxed” *Ent1* allele with transgenic mice that express Cre-recombinase under the control of a cardiac myocyte specific promoter (*Ent1^loxP/loxP^* Myosin Cre^+^ mice) (Figure 3A). Real-time PCR results demonstrated significantly diminished expression of *Ent1* mRNA in isolated cardiomyocytes in *Ent1^loxP/loxP^* Myosin Cre^+^ mice compared with Myosin Cre^+^ control mice, while *Ent2* transcript level was unchanged (Figure 3B). Subsequent Western blot experiments showed similar alterations in protein levels of ENTs (Figure 3, C and D). In contrast, the ENT1 transcript and protein levels remained essentially unchanged in other cell types within the heart, such as cardiac endothelial cells and cardiac fibroblasts, as well as in other tissues, including lungs and kidneys, in *Ent1^loxP/loxP^* Myosin Cre^+^ mice (Supplemental Figure 1; supplemental material available online with this article; https://doi.org/10.1172/jci.insight.166011DS1). Next, we exposed *Ent1^loxP/loxP^* Myosin Cre^+^ mice to myocardial IRI and discovered similar phenotypes that we previously identified in *Ent1^–/–^* mice, including smaller infarct sizes (Figure 3, E and F) and attenuated serum troponin I levels (Figure 3G). Subsequently, we also generated *Ent2^loxP/loxP^* Myosin Cre^+^ mice using a similar approach (17, 18) and exposed them to myocardial IRI (Figure 4A). We first confirmed the specific knockdown effect in *Ent2* transcript (Figure 4B) and protein levels (Figure 4, C and D) in isolated cardiac myocytes in *Ent2^loxP/loxP^* Myosin Cre^+^ mouse hearts (Supplemental Figure 2). Our study also examined the impact of myocyte-specific *Ent2* deletion on myocardial injury. Interestingly, *Ent2^loxP/loxP^* Myosin Cre^+^ mice exhibited similar myocardial injury compared with control mice, as demonstrated by comparable infarct sizes (Figure 4, E and F) and troponin I levels (Figure 4G). Furthermore, our findings exhibit congruity with previous research conducted on HL-1 cells, a murine cardiomyocyte cell line, that indicates that ENT1 accounts for 85% of adenosine transport, with a mere 15% being attributed to ENT2 (32). These findings align with our findings, which show that *Ent1^loxP/loxP^* Myosin Cre^+^ mice exhibited dampened myocardial injury than *En21^loxP/loxP^* Myosin Cre^+^ mice. Our results provide further support for the notion that myocyte ENT1 is the dominant nucleoside transporter in the heart and that its downregulation could be a promising strategy for limiting myocardial injury in the context of ischemic heart disease.

### ENT inhibition or deletion extends cardiac adenosine elevations throughout reperfusion.

**Figure 5:**
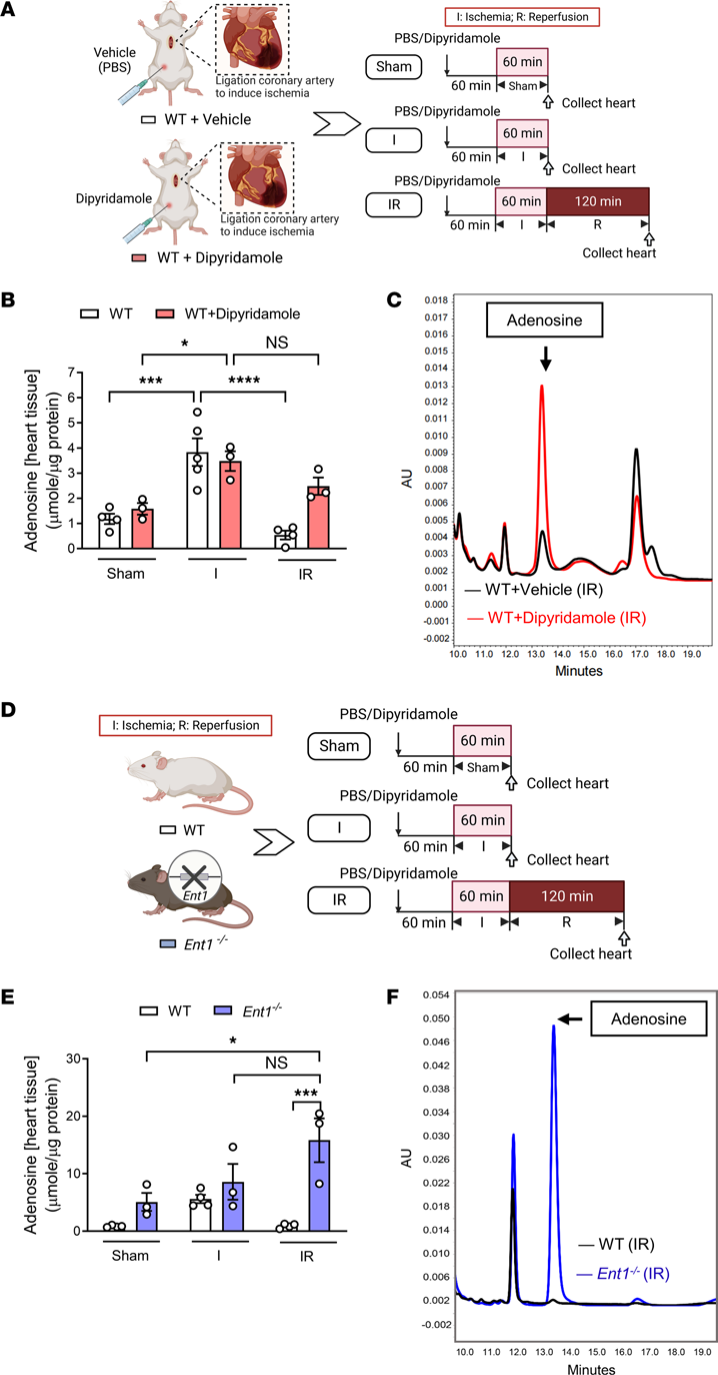
ENT inhibition extends cardiac adenosine elevations throughout reperfusion. (**A**) C57BL/6J mice received either PBS or dipyridamole (5 mg/kg, i.p.) 1 hour before being exposed to 1 of the 3 conditions: sham, 60 minutes of ischemia (I group), or 60 minutes of ischemia followed by 2 hours of reperfusion (IR group). (**B**) Cardiac adenosine levels were measured from the area at risk by high-performance liquid chromatography (HPLC) (*n* = 3–5, 2-way ANOVA, *P < 0.05, ***P < 0.001, ****P < 0.0001 in Bonferroni’s multiple-comparison test). (**C**) Representative cardiac adenosine levels in the IR group in WT mice treated with PBS or dipyridamole. (**D**) WT or *Ent1^–/–^* mice were exposed to sham, 60 minutes of ischemia (I group), or 60 minutes of ischemia and 2 hours of reperfusion (IR group). (**E**) Cardiac adenosine levels were measured from the area at risk by HPLC (*n* = 3–4, 2-way ANOVA, *P < 0.05, ***P < 0.001 in Bonferroni’s multiple-comparison test). (**F**) Representative cardiac adenosine levels in the IR group in WT or *Ent1^–/–^* mouse hearts. Data are shown as mean ± SEM. Each dot represents 1 mouse.

After having demonstrated a selective role for myocyte-specific *Ent1*, we next pursued studies on the functional roles of ENTs in modulating cardiac adenosine levels. We used high-performance liquid chromatography (HPLC) to assess cardiac adenosine levels, as previously described (19). In these studies, we treated mice with vehicle or dipyridamole (5 mg/kg, i.p.) 60 minutes prior to the onset of myocardial ischemia (Figure 5A). We subsequently collected left ventricular tissues from the AAR in sham-operated mice, after 60 minutes of ischemia (I group), or after 60 minutes of ischemia followed by 2 hours of reperfusion (IR group). We found significant increases in cardiac adenosine levels after 60 minutes of exposure to myocardial ischemia in both vehicle-treated or dipyridamole-treated mice (Figure 5B). Importantly, our study revealed that elevations of cardiac adenosine levels returned to baseline after 2 hours of reperfusion in vehicle-treated mice, whereas mice pretreated with dipyridamole experienced persistent elevations of cardiac adenosine throughout reperfusion (Figure 5, B and C). Next, we pursued similar studies in gene-targeted mice for *Ent1* (*Ent1^–/–^* mice; Figure 5D). Consistent with the pharmacologic studies above, we found that genetic deletion of *Ent1* was associated with a considerably heightened concentration of cardiac adenosine throughout reperfusion (Figure 5, E and F). Taken together, these studies demonstrate that targeting ENT1 is associated with persistent elevations of cardiac adenosine levels throughout reperfusion.

### ENT-dependent cardioprotection involves myeloid-expressed Adora2b receptors.

**Figure 6:**
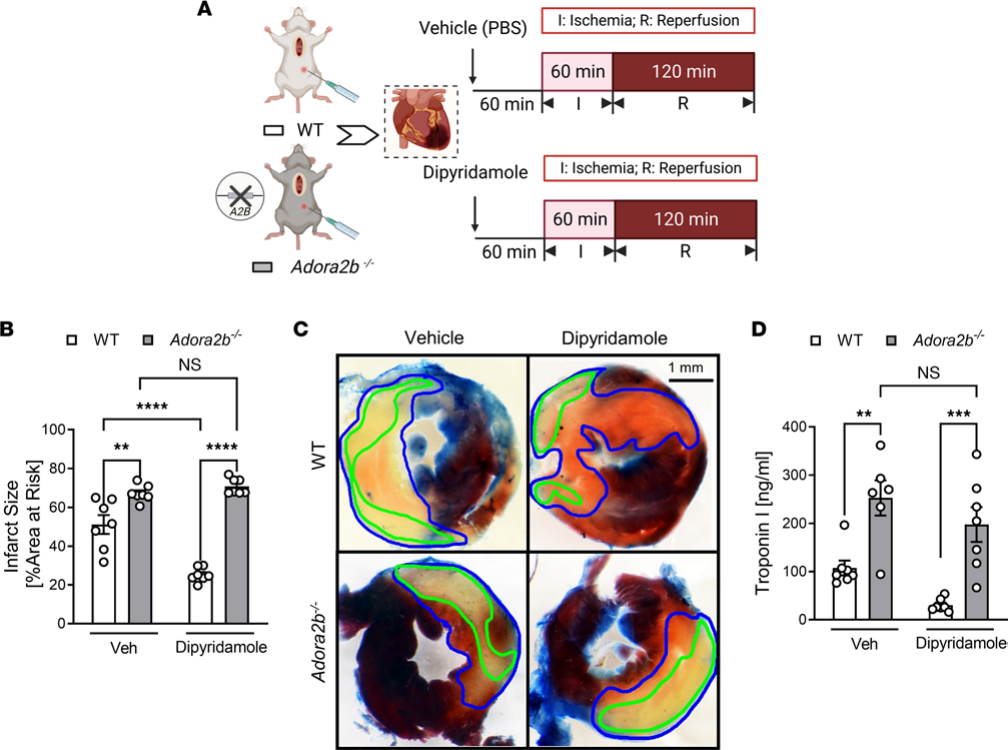
Global *Adora2b* whole-body KO dampens the cardioprotective effects of dipyridamole treatment. (**A**) WT control and *Adora2b^–/–^* mice treated with PBS or dipyridamole (5 mg/kg, i.p.) were exposed to in situ myocardial IRI. (**B**) Infarct sizes in WT control and *Adora2b^–/–^* mice (*n* = 7; 2-way ANOVA, **P < 0.01, ****P < 0.0001 in Bonferroni’s multiple-comparison test). (**C**) Representative images of infarct staining. The infarct area is outlined by a green line; AAR is outlined by a blue line. Scale bar: 1 mm. (**D**) cTnI levels after surgery (*n* = 7; 2-way ANOVA, **P < 0.01, ***P < 0.001 in Bonferroni’s multiple-comparison test). Data are shown as mean ± SEM. Each dot represents 1 mouse.

**Figure 7:**
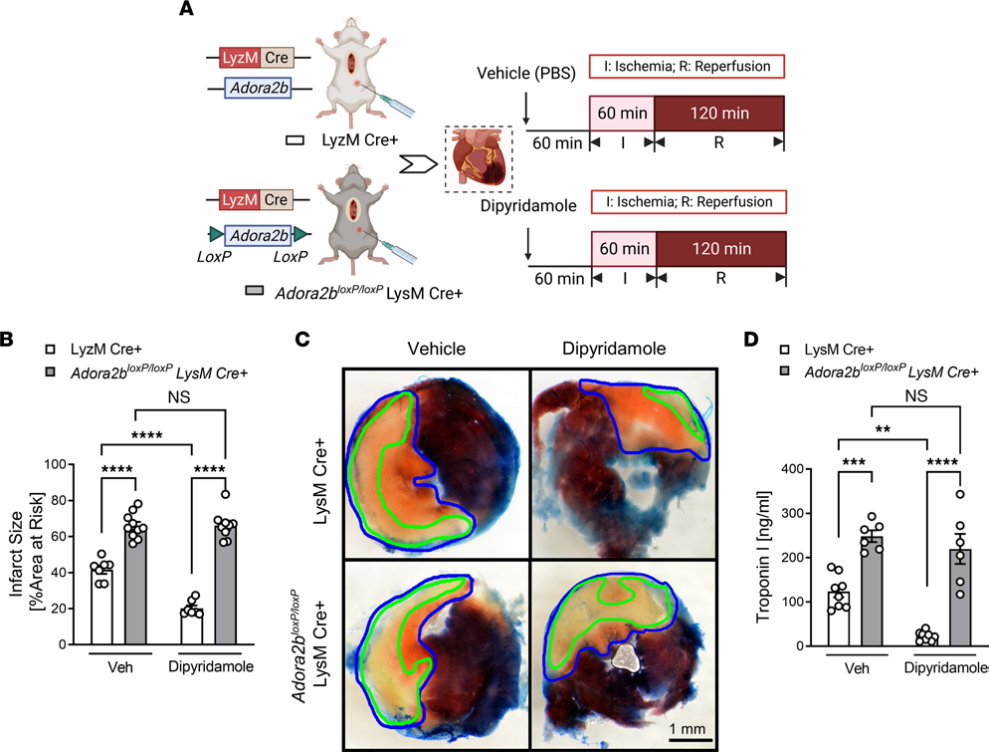
Myeloid *Adora2b* deletion dampens the cardioprotective effects of dipyridamole treatment. (**A**) LysM Cre^+^ and *Adora2b^loxP/loxP^* LysM Cre^+^ mice treated with PBS or dipyridamole (5 mg/kg, i.p.) were exposed to in situ myocardial IRI. (**B**) Infarct sizes in LysM Cre^+^ and *Adora2b^loxP/loxP^* LysM Cre^+^ mice (*n* = 7–10; 2-way ANOVA, ****P < 0.0001 in Bonferroni’s multiple-comparison test). (**C**) Representative images of infarct staining. The infarct area is outlined by a green line; AAR is outlined by a blue line. Scale bar: 1 mm. (**D**) cTnI levels after surgery (*n* = 7–8; 2-way ANOVA, **P < 0.01, ***P < 0.001, ****P < 0.0001 in Bonferroni’s multiple-comparison test). Data are shown as mean ± SEM. Each dot represents 1 mouse.

We next pursued studies to identify relevant adenosine receptors in mediating the observed cardioprotection. We first focused on Adora2b since several previous studies highlight a specific role for Adora2b signaling in cardioprotection (4, 22). For this purpose, we administered dipyridamole (5 mg/kg, i.p.) or vehicle control (PBS) to control animals (C57BL/6J mice) and mice with global *Adora2b* deletion (*Adora2b^–/–^* mice) (18, 22, 29), and we subjected them to in situ myocardial IRI (Figure 6A). Our results indicate that myocardial injury levels were attenuated in WT controls treated with dipyridamole, compared with vehicle, but that the protective effects of dipyridamole were abolished in *Adora2b^–/–^* mice (Figure 6, B–D). Based on previous studies implicating myeloid-expressed Adora2b signaling in attenuating myocardial reperfusion injury (33), we next repeated similar studies (Figure 7A) in *Adora2b^loxP/loxP^* LysM Cre^+^ mice (29, 34). We first confirmed the specific knockdown of Adora2b at both the transcript and protein levels in myeloid cells in *Adora2b^loxP/loxP^* LysM Cre^+^ mice compared with LysM Cre^+^ mice (Supplemental Figure 3). Subsequently, we observed that dipyridamole treatment did not confer protection to *Adora2b^loxP/loxP^* LysM Cre^+^ mice against myocardial IRI, as evidenced by the lack of significant difference in myocardial infarct sizes (Figure 7, B and C) and serum troponin I levels (Figure 7D), when compared with LysM Cre^+^ mice. Taken together, these findings highlight a functional role of myeloid-expressed Adora2b signaling in mediating ENT inhibitor–elicited protection from myocardial IRI.

## Discussion

**Figure 8:**
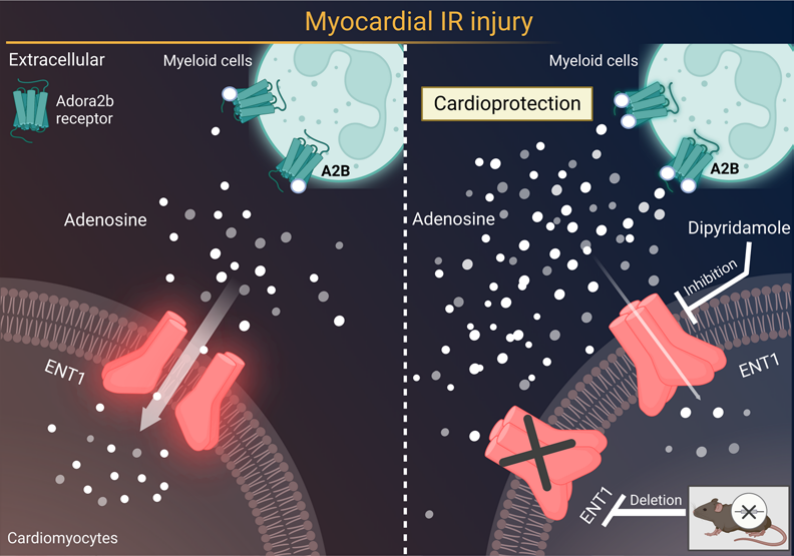
Targeting myocardial ENT1 provides cardioprotection by enhancing myeloid Adora2b signaling. Previous studies implicate adenosine signaling in attenuating myocardial ischemia and reperfusion injury. Here, we show that pharmacologic inhibition or genetic deletion of *Ent1* enhances extracellular adenosine signaling during myocardial IRI, ultimately promoting cardioprotection through myeloid Adora2b adenosine receptors. Gray arrow indicates the direction of the adenosine flow. A2B, Adora2b adenosine receptor.

Finding novel approaches to prevent or treat myocardial injury caused by ischemia and reperfusion is of very high interest (3, 35–38). Particularly, pharmacologic strategies that would dampen myocardial reperfusion injury are highly desirable due to their potential impact for translation into clinical practice in patients with myocardial IRI or cardiac surgery (10, 39, 40). In the present study, we examined the molecular mechanisms responsible for the termination of extracellular adenosine signaling during myocardial injury to identify targeting strategies that would allow for the enhancement of extracellular adenosine signaling to promote cardioprotection. Initial pharmacologic studies with the nonspecific ENT inhibitor dipyridamole revealed attenuation of myocardial infarct sizes and troponin levels. Subsequent studies in genetic models of global and tissue-specific deletion of ENTs pointed toward a role of myocyte-expressed ENT1. *Ent1^loxP/loxP^* Myosin Cre^+^ mice experienced attenuated myocardial infarct sizes. In contrast, *Ent2^loxP/loxP^* Myosin Cre^+^ mice had similar cardiac injury after 60 minutes of ischemia and 2 hours of reperfusion compared with Myosin Cre^+^ controls. Subsequent studies demonstrated that targeting ENTs was associated with elevated cardiac adenosine levels throughout reperfusion, thus implicating adenosine signaling in the associated cardioprotection. In addition, studies using dipyridamole treatment in gene-targeted mice for adenosine receptors revealed a functional role for myeloid Adora2b signaling since cardioprotective effects of dipyridamole were completely abolished in *Adora2b^loxP/loxP^* LysM Cre^+^ mice (Figure 8). Taken together, the present studies implicate ENT inhibitors in cardioprotection by attenuating reperfusion injury and provide insights into a molecular crosstalk pathway between myocardial ENT1 and myeloid Adora2b adenosine receptors during cardioprotection.

Consistent with our findings, other studies also demonstrated the functional roles of ENTs expressed on RBCs as a critical component for terminating intravascular adenosine levels, particularly following i.v. adenosine injection (16). I.v. adenosine is used as mainstay therapy for the treatment of supraventricular tachycardia (11, 41). The elevations of extracellular adenosine following i.v. bolus injection can lead to dramatic increases in extracellular adenosine (42). Increased adenosine and subsequent activation of the Adora1 mediates a complete heart block that lasts approximately 5–10 seconds (41, 43). Mice with genetic deletion of the *Adora1* are resistant to the heart rate–slowing effects of intravascular adenosine bolus treatment (11). Notably, ENTs expressed on erythrocytes are assumed to be responsible for adenosine uptake after i.v. injection and the short half-life of extracellular adenosine in this context (44). A recent study implicates ENT-dependent adenosine uptake on erythrocytes in high-altitude adaptation (45). This study demonstrates that plasma adenosine levels of volunteers exposed to high altitude are elevated and more rapidly elevated after descent and reascent. This observation strongly implicates this hypoxic adenosine response in acclimatization and adaptation during high altitude exposure (45). Subsequent molecular studies in blood samples derived from the volunteers in this study found repressed levels of erythrocyte-dependent ENT1 (45). Studies in mice with deletion of *Ent1* on erythrocytes (*Ent1^loxP/loxP^* EpoRCre^+^ mice) provide evidence that these animals experience attenuated tissue hypoxia and inflammation during 72 hours of hypoxia exposure; this serves as an explanation for the above observations in climbers (45). Other studies implicate erythrocyte-dependent Adora2b signaling as a critical step in hypoxia adaptation (46, 47). It is conceivable that ENT1 expressed on erythrocytes could also play functional roles in cardioprotection. However, the fact that tissue-specific *Ent1* deletion on myocytes (*Ent1^loxP/loxP^* Myosin Cre^+^ mice) mirrors the cardioprotective phenotype of global *Ent1* deletion argues for a predominant role of myocyte-specific adenosine during myocardial injury.

Adenosine receptors have been previously implicated in cardioprotection during ischemia and reperfusion. For example, previous studies demonstrate that Adora2a is expressed in immune cells (10) and functions to dampen excessive inflammation (9, 48, 49). Similar studies provide evidence that the Adora2a expression on CD4^+^ T cells contributes to attenuated myocardial IRI in murine models (50). Previous studies of Adora2b signaling during myocardial IRI have shown that mice with global *Adora2b* deletion (*Adora2b^–/–^* mice) experienced larger infarct sizes and abolished cardioprotection provided by ischemic preconditioning (23). Moreover, the Adora2b agonist BAY 60-6583 was found to dampen myocardial infarct sizes in mouse (22, 23) or rat (51) models of myocardial IRI. Other studies implicate Adora2b signaling in altering the immune cell composition and enhancement of IL-6 release, thereby promoting remodeling after ischemic injuries (52). Moreover, adenosine has been shown to form a negative feedback loop that inactivates platelet aggregation and inflammatory interactions with immune cells such as monocytes and neutrophils via binding to the Adora2a (A2A) and Adora2b (A2B) receptors on platelets, ultimately leading to decreased formation of platelet-neutrophil complexes and attenuation of ischemic inflammatory injury (53). Recent research has elucidated the significant functional contributions of Adora2b in the normoxic induction of hypoxia-inducible factor HIF1A and the generation of epicardial stromal cells following myocardial infarction (54). Further mechanistic investigations have revealed that a group of signaling molecules known as neuronal guidance proteins (NGPs) — which are secreted by various tissues such as endothelial cells, immune cells, RBCs, and cardiomyocytes — play a significant role in the regulation of tissue inflammation and immune cell activation (55, 56). Our recent study directly implicates myeloid-expressed Adora2b signaling in attenuating myocardial reperfusion injury through a crosstalk pathway with a neuronal guidance molecule netrin-1 (29). This study demonstrates that patients with myocardial IRI experienced elevated netrin-1 levels. Subsequent laboratory studies showed that polymorphonuclear leukocytes (PMN) are the source of netrin-1 elevations during myocardial injury. Mice with myeloid netrin-1 deletion (*Ntn1^loxP/loxP^* LysM Cre^+^) had larger myocardial infarct sizes, and therapeutic effects of treatment with recombinant netrin-1 were abolished in *Adora2b^loxP/loxP^* LysM Cre^+^ mice (29). Consistent with other studies (33, 57, 58), these findings highlight a critical role of Adora2b signaling in attenuating myocardial injury. While the studies of netrin-1–dependent Adora2b signaling highlight an autocrine pathway (29), the present studies provide evidence for a crosstalk between myocyte *Ent1* and myeloid Adora2b.

### Conclusion.

The present findings demonstrate a previously unrecognized role of cardiac myocyte-expressed ENT1 in terminating extracellular adenosine signaling during myocardial IRI. Pharmacologic studies using ENT inhibitors and genetic studies with global or myocyte-specific *Ent1* deletion provide evidence for the enhancement of extracellular adenosine signaling in conjunction with cardioprotection and implicate myeloid Adora2b signaling in mediating this protection. Future studies should explore the possibility of utilizing these findings for translational studies, by using global or specific ENT inhibitors to treat or prevent myocardial injury in patients with myocardial ischemia and reperfusion or during cardiac surgery.

## Methods

### Mice.

Regarding sex as a biological variable, we conducted myocardial ischemia and reperfusion surgery in both male and female mice aged between 8 and 16 weeks. A sex-specific analysis regarding mouse infarct size and troponin levels was performed, and no significant differences between sexes were found. WT C57BL/6J (The Jackson Laboratory, 000664), CMV Cre^+^ (B6.C-Tg[CMV Cre]1Cgn/J, The Jackson Laboratory, 006054), Myosin Cre^+^ (STOCK A1cf^Tg[Myh6–cre/Esr1*]1Jmk^/J, The Jackson Laboratory, 005650) (59), and LysM Cre^+^ (B6.129P2-*Lyz2^tm1[cre]Ifo^*/J, The Jackson Laboratory, 004781) (60) mice were purchased from The Jackson Laboratory. *Ent1*-deficient mice were provided by Doo-Sup Choi (The Mayo Clinic, Rochester, Minnesota, USA) (31), and C57BL/6J mice were used as control. *Ent1* floxed (*Ent1^loxP/loxP^*) mice, *Ent2* floxed (*Ent2^loxP/loxP^*) mice, and *Adora2b* floxed (*Adora2b^loxP/loxP^*) mice (33) were generated by Ozgene. *Ent2^−/−^* mice were generated by crossbreeding *Ent2^loxP/loxP^* mice with CMV Cre^+^, and C57BL/6J mice were used as control. *Ent1^loxP/loxP^* Myosin Cre^+^ and *Ent2^loxP/loxP^* Myosin Cre^+^ mice were obtained by crossbreeding floxed mice with Myosin Cre^+^ mice, and Myosin Cre^+^ mice were used as control. *Adora2b^−/−^* mice (17) were generated by crossbreeding *Adora2b^loxP/loxP^* mice with CMV Cre^+^, and WT mice were used as control. *Adora2b^loxP/loxP^* LysM Cre^+^ (29, 34) were obtained by crossbreeding floxed mice with LysM Cre^+^, and LysM Cre^+^ was used as a control. To induce the Cre recombinase in Myosin Cre^+^ mouse hearts, mice were injected with tamoxifen (Cayman, 13258), 50 μg/g, i.p., for 5 consecutive days (29, 61–63). Animals were genotyped, and knock-down efficiencies were confirmed as we have done previously (61, 63). To minimize animal numbers, the sample sizes were calculated based on pilot studies and power analysis (22, 61, 63).

### A murine model for myocardial IRI.

An in situ murine model of myocardial IRI was established as we have done previously (28, 29, 61, 63). In short, after anesthesia induction by pentobarbital sodium (70 mg/kg, i.p., McKesson, 809790), intubation and ventilation were conducted with pressure-controlled ventilation (frequency, 110 breaths per min; peak inspiratory pressure, 14 cm H_2_O; positive end-expiratory pressure, 4 cm H_2_O with FiO_2_ of 0.4). Heart rate was monitored with an ECG. Mouse body temperature was maintained at 37°C during surgical procedures using a temperature-controlled surgical table with a rectal thermometer. The optimal fluid infusion was obtained via a catheter in the carotid artery. From that point forward, all operations were performed under a research stereomicroscope system SZX10 (Olympus). The mouse heart was nicely exposed, and the left main coronary artery was ligated with an 8-0 suture (Prolene, EP8732H). Successful occlusion was confirmed by the color change of the left ventricular wall (from bright red to pale). In addition, an ST-elevation should also be observed by ECG. After 60 minutes of ischemia, reperfusion persisted for another 2 hours and was confirmed by ECG (resolution of ST-segment elevation) and visual inspection (return of coloration and movement). In the sham surgery group, mice underwent a similar procedure without left main coronary artery occlusion.

### Dipyridamole treatment.

Dipyridamole solution is prepared as follows: 20 mg dipyridamole (Sigma-Aldrich, D9766) was first diluted in 400 μL of DMSO (Cayman, 71210) and then in 2 mL of 100% ethanol. Sonication was performed in a water bath until it was completely dissolved. Finally, 17.6 mL of corn oil was added to the solution for a final working concentration of 1 mg/mL (19). The solution was kept in the dark due to light sensitivity. Mice were pretreated with dipyridamole, 5 mg/kg, i.p. 60 minutes prior to the onset of myocardial ischemia. The control groups were given the same amount of PBS.

### Assessment of infarct size.

Infarct size analysis was calculated by the percentage of myocardium infarcted within the AAR, as we have done previously (29, 61). After 2 hours of reperfusion, the heart was flushed using 0.9% saline and then injected with 800 μL of Evans blue (1%, Sigma-Aldrich, E2129) via the carotid artery catheter, followed by permanent left coronary occlusion. The heart was collected and cut with a microtome (Roboz, SA-4130) at a thickness of 1 mm. The slices were double-stained using 1% TTC (Sigma-Aldrich, T8877) for 10 minutes in the water bath at 37°C and then fixed in 10% formalin overnight. TTC-stained heart slices were photographed and analyzed using ImageJ (NIH).

### Cardiac troponin I ELISA.

Serum was collected immediately after the surgery via the inferior vena cava. Myocardial injury was evaluated by serum troponin I level via ELISA by using the cardiac troponin I ELISA Kit (Life Diagnostics, CTNI-1-HS) as we have done previously (29, 63).

### Adult murine cardiomyocyte isolation.

Cardiac myocytes were isolated according to the protocol, as previously done (33, 64). Briefly, mouse hearts were collected after being flushed with 0.9% saline and EDTA buffer via a carotid artery catheter after euthanization. Then, the left ventricles were minced into 1–2 mm cubes and incubated with collagenase buffer. The rest digestion steps were performed using a gentleMACS Octo Dissociator with Heaters (Miltenyi Biotec), and the cardiomyocytes were obtained by passing the cell suspension through a 100 μm strainer.

### Adult murine cardiac endothelial cell isolation.

Cardiac endothelial cells were isolated according to the protocol, as we have done previously (33). In brief, the heart tissue was processed by initially performing transcardial perfusion using ice-cold PBS (Thermo Fisher Scientific), followed by cutting the heart into small pieces and placing them in a 50 mL conical tube containing 5 mL of heart digestion buffer (supplemented DMEM combined with 0.1% collagenase II, 0.25% collagenase IV, and 7.5 μg/mL DNAse I [all from Thermo Fisher Scientific]). The sample was then incubated at 37°C for 25 minutes and dissociated using the gentleMACS Dissociator in a gentleMACS C tube. According to the manufacturer’s protocol, the resulting cell suspension is enriched for endothelial cells using CD31 murine MicroBeads (Miltenyi Biotec, 130-097-418). The enriched cells are then stained with CD31 FITC (Thermo Fisher Scientific, 11-0311-82; 1:300), CD45 PE-Cyanine7 (Thermo Fisher Scientific, 25-0451-82; 1:700), and eFluor 450 viability dye (Thermo Fisher Scientific, 65-0863-18; 1:1000), followed by centrifugation at 300*g* for 5 minutes at 4**°**C and resuspension in wash buffer for FACS analysis. Finally, viable CD45^–^ and CD31^+^ cells were sorted and collected in tubes (65).

### Adult murine cardiac fibroblasts isolation.

Mouse hearts were collected and enzymatically dissociated to obtain a single-cell suspension for flow cytometry analysis. The cell pellet was resuspended in FACS buffer (PBS supplemented with 1% FBS and 1 mM EDTA) to obtain a single-cell suspension and subsequently blocked with anti–mouse CD16/CD32 (1:200; Thermo Fisher Scientific, 14-0161-82) for 10 minutes at 4°C. The cells were then incubated on ice for 30 minutes with a suitable combination of fluorochrome-conjugated antibodies diluted in FACS buffer: TER119 PE (eBioscience, 11-5921-82), CD45 PE (1:300, eBioscience, 14-0451-82), CD31 PE (1:300, Thermo Fisher Scientific, 14-0311-82), and gp38 APC (eBioscience, 14-5381-82). Live/dead cells were distinguished using DAPI (1 μg/mL). Sorted lineage^–^ (Ter119^−^CD45^−^CD31^−^) and gp38^+^ cells were cultured and induced to differentiate with 10 ng/mL mouse recombinant TGF-β1 (BioLegend, 763104) (66).

### Adult murine BM myeloid cells isolation.

Mouse BM myeloid cells were isolated according to the protocol, as we have done previously (33). First, to obtain BM cells, the femur and tibia were flushed using a syringe equipped with a 23-gauge needle, and the resulting cell suspension was collected in the appropriate medium. The suspension was then gently passed through the syringe several times to disperse any clumps. A 70 μm mesh nylon strainer was used to eliminate any remaining clumps or debris from the cell suspension. After resuspension, myeloid cells were isolated using EasySep Mouse CD11b Positive Selection Kit II (StemCell Technologies, 18970), according to the manufacturer’s instructions.

### Murine cardiac adenosine measurement.

Nucleosides were extracted from the left ventricles of mice using the protocatechuic acid (PCA) extraction procedure (29). Briefly, mouse left ventricles were homogenized in PBS on ice in the presence of the protease inhibitor cocktail (Thermo Fisher Scientific) and nucleosides preserve cocktail (10 μM dipyridamole, 10 μM deoxycoformycin, and 10 μM αβ-methylene ADP [all from Sigma-Aldrich]). Homogenized samples were centrifuged at 12,000*g* at 4**°**C for 5 minutes, and the protein concentrations were determined in the supernatant. The supernatant was then mixed with 0.4N PCA to precipitate the proteins. Samples were then neutralized with KHCO_3_/KOH, followed by acidification with NH_4_PO_4_ and phosphoric acid. The supernatant was obtained after centrifugation at 17,000*g* at 4**°**C for 10 minutes and analyzed by HPLC as described previously (17, 19). Adenosine peaks were detected, and calibration curves were obtained using external standard curves. Data were normalized to the total protein amount of the heart.

### Transcriptional analysis.

Total RNA was extracted from the tissues and isolated cells using RNeasy mini kit (Qiagen, 74106), according to the manufacturer’s instructions, as we have done previously (17, 29, 63). In short, cDNA was synthesized using the High-Capacity cDNA Reverse Transcription Kit (Thermo Fisher Scientific, 4368814). The real-time PCR reactions were performed with SYBR Green (Qiagen, 204145) on the Bio-Rad CFX384 Touch Real-Time PCR Detection System with the following primers: mouse *Ent1* forward: 5′- CTT GGG ATT CAG GGT CAG AA-3′, mouse *Ent1* reverse: 5′- ATC AGG TCA CAC GAC ACC AA -3′; mouse *Ent2* forward: 5′- CAT GGA AAC TGA GGG GAA GA -3′, mouse *Ent2* reverse: 5′- GTT CCA AAG GCC TCA CAG AG-3′; mouse *Adora2b* forward: 5′-GTG GGG GTC TGT AAT GCA CT-3′; mouse *Adora2b* reverse: 5′-AGC TAG AGA CGC AAG ACG C-3′; mouse β-actin forward: 5′- GGCTGTATTCCCCTCCATCG -3′; and mouse β-actin reverse: 5′- CCAGTTGGTAACAATGCCATGT -3′. Relative quantification was performed using the comparative Ct method, and the data were illustrated as the mean ratio to β-actin.

### Western blot.

Mouse tissues or isolated cells were lysed and extracted in RIPA lysis buffer (Thermo Fisher Scientific, 89900) with both protease (Thermo Fisher Scientific, 78425) and phosphatase inhibitor (Thermo Fisher Scientific, 78420) cocktail added. In total, 10–20 μg of protein was then separated by SDS-PAGE and immunoblotted with the following primary antibodies: primary rabbit anti-ENT1 (Abcam, 48607), rabbit anti-ENT2 (Abcam, 48595), rabbit anti-ADORA2B (Abcam, 229671), or mouse anti–α-tubulin antibodies (Cell Signaling, 3873) and corresponding secondary antibodies conjugated to horseradish peroxidase: anti-rabbit (Cell Signaling Technology, 7074), and anti-mouse (Cell Signaling Technology, 7076). Finally, the membranes were developed using the appropriate substrate, and the signal intensity was measured using ImageJ (NIH). Results were normalized using α-tubulin and expressed as relative fold change.

### Statistics.

All data are expressed as mean ± SEM, and statistical analysis was performed with GraphPad Prism 9.0. Robust nonlinear regression method was used to identify outliers. Here, no outlier was detected. Shapiro-Wilk normality test was conducted to evaluate normal distributions. If the normality assumption held, a 2-tailed unpaired *t* test was used for the equal variances assumed (accessed by F test), or 2-tailed Welch’s *t* tests were performed when variances were unequal. If the normality assumption didn’t hold, we used a Mann-Whitney *U* test to compare medians. In the case of more than 2 groups, the Brown-Forsythe test was conducted to check equal variances. Thereafter, 1-way ANOVA or 2-way ANOVA, followed by the Bonferroni post hoc test, was used to determine the significance. *P* < 0.05 was considered significant.

### Study approval.

All procedures involving animal experiments were approved by the University of Texas Health Science Center at Houston IACUC.

## Author contributions

TWM and HKE designed the research studies. WR, JL, and SC performed the experiments. WR and JL acquired the data and conducted the data analyses. WR and HKE wrote the manuscript. RN, XM, YL, and XY edited the manuscript.

## Supplementary Material

Supplemental data

## Version 1 06/08/2023

Electronic publication
