# Supplementary material for: Targeting myocardial equilibrative nucleoside transporter ENT1 provides cardioprotection by enhancing myeloid Adora2b signaling
Source: JCI Insight. 2023 Jun 8;8(11):e166011. doi: 10.1172/jci.insight.166011 (PMC10393224; doi:10.1172/jci.insight.166011)
Supplement: Supplemental data [file jciinsight-8-166011-s053.pdf]

Supplemental Figures

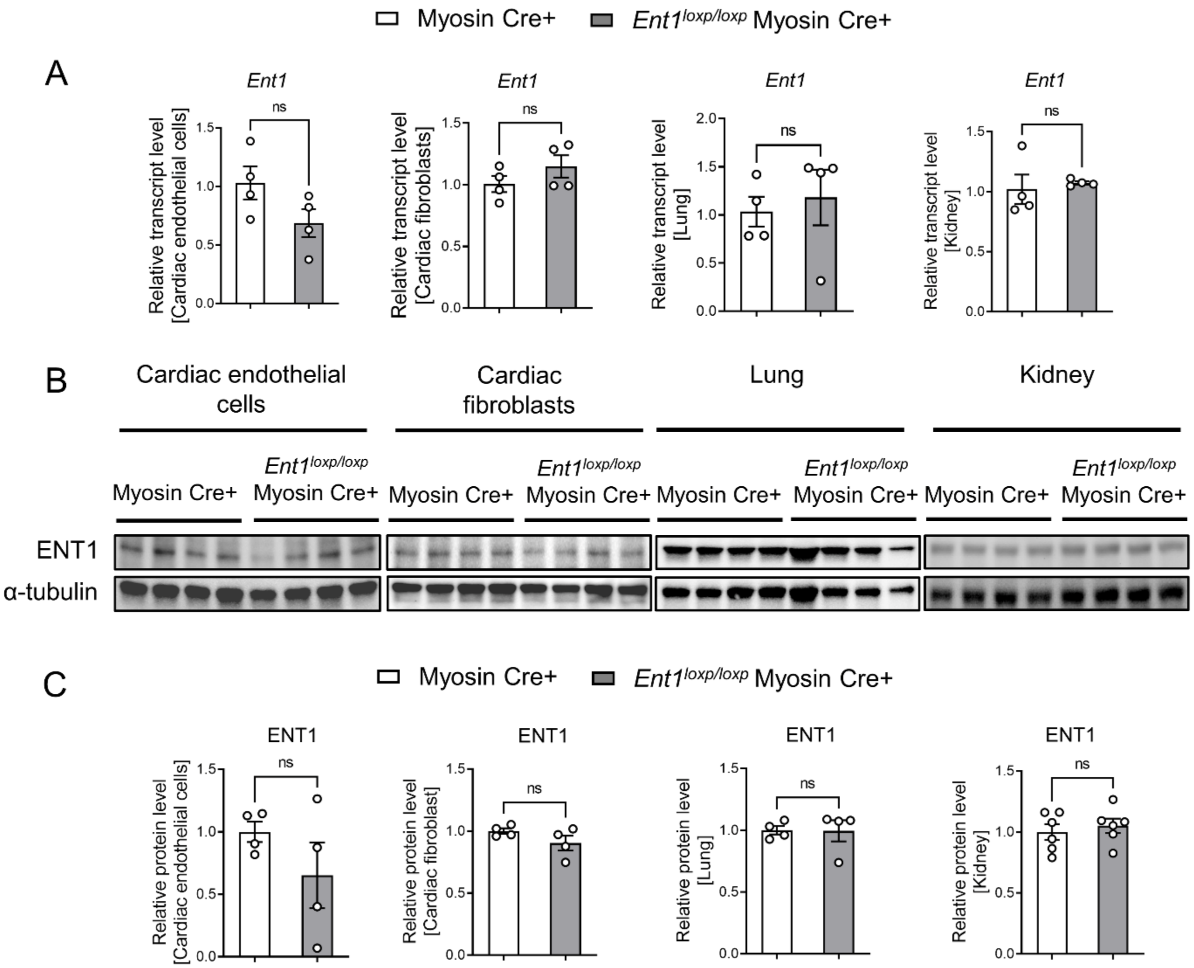

Supplemental Figure 1. Tissue-specific deletion of *Ent1* in *Ent1*$^{loxp/loxp}$ Myosin Cre+ mice. **(A)** *Ent1* transcript levels in cardiac endothelial cells, cardiac fibroblasts, lungs, and kidneys from Myosin Cre+ or *Ent1*$^{loxp/loxp}$ Myosin Cre+ mice (n = 4; two-tailed unpaired t-test for cardiac endothelial cells, cardiac fibroblasts, and kidney samples; Mann-Whitney test for lung samples). **(B)** ENT1 protein levels by western blot analysis. **(C)** Quantification of **(B)** (n = 4-6; two-tailed unpaired t-test for cardiac endothelial cells, cardiac fibroblasts, and kidney samples; Welch's test for lung samples). Values are mean ± SEM. Each dot represents 1 mouse.

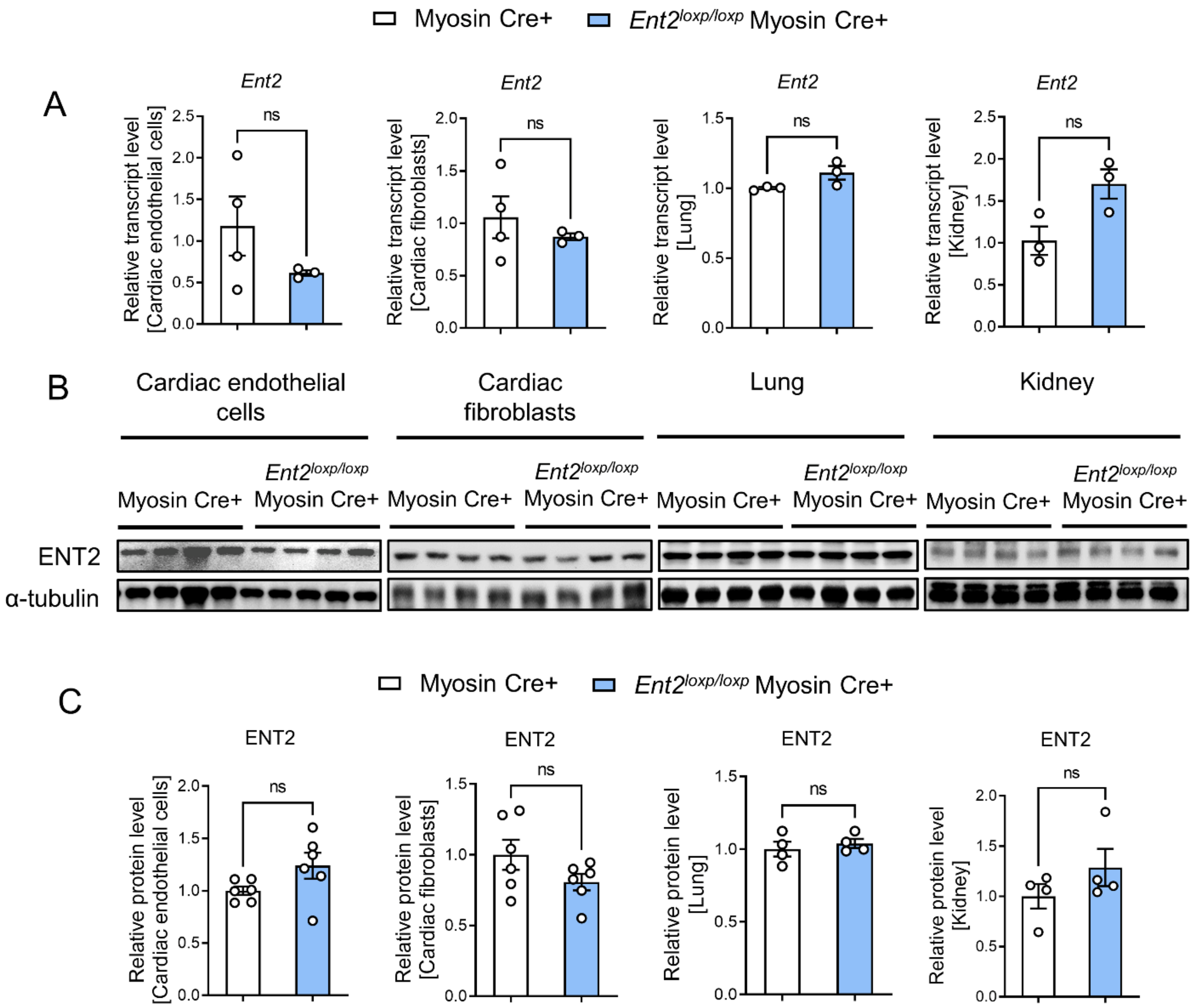

Supplemental Figure 2. Tissue-specific deletion of *Ent2* in *Ent2*$^{loxp/loxp}$ Myosin Cre+ mice. **(A)** *Ent2* transcript levels in cardiac endothelial cells, cardiac fibroblasts, lungs, and kidneys from Myosin Cre+ or *Ent2*$^{loxp/loxp}$ Myosin Cre+ mice (n = 3-4; Welch's test for cardiac endothelial cells and cardiac fibroblasts; two-tailed unpaired t-test for lung and heart samples). **(B)** ENT2 protein levels by western blot analysis. **(C)** Quantification of **(B)** (n = 4-6; Welch's test for cardiac endothelial cells and kidney samples; two-tailed unpaired t-test for cardiac fibroblasts and lung samples). Values are mean ± SEM. Each dot represents 1 mouse.

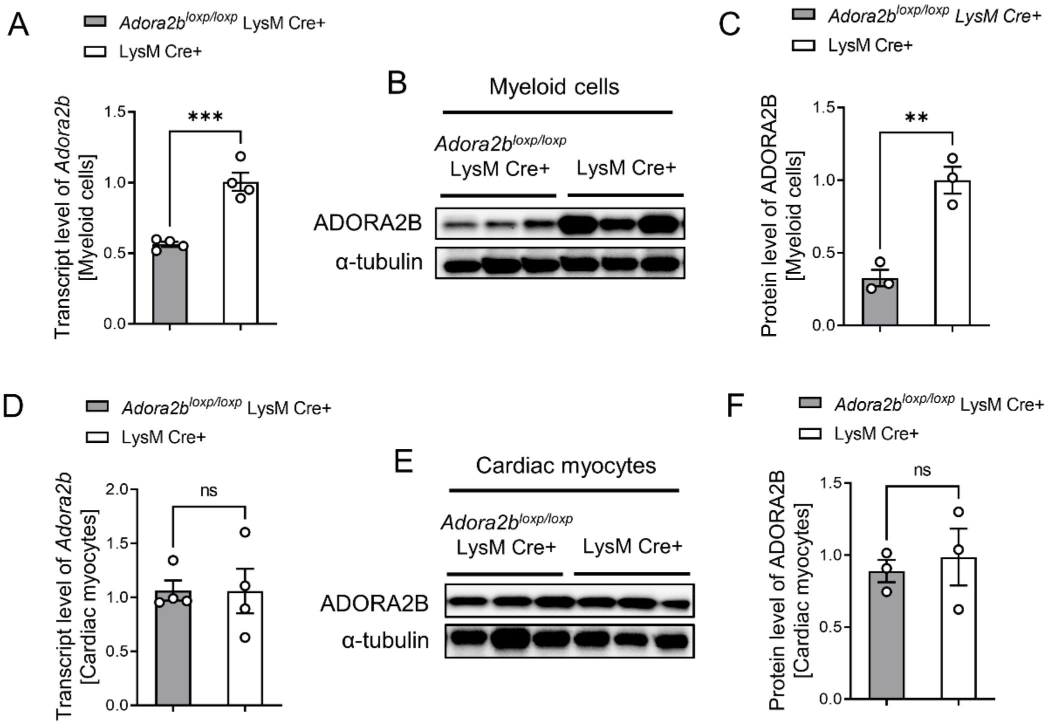

Supplemental Figure 3. Tissue-specific deletion of *Adora2b* in *Adora2b$^{loxp/loxp}$* LysM+ mice. **(A)** *Adora2b* transcript levels in isolated bone marrow myeloid cells from *Adora2b$^{loxp/loxp}$* LysM+ mice or LysM+ mice (n = 4; two-tailed unpaired t-test). **(B)** ADORA2B protein levels by western blot analysis. **(C)** Quantification of **(B)** (n = 3; two-tailed unpaired t-test). **(D)** *Adora2b* transcript levels in isolated cardiomyocytes from *Adora2b$^{loxp/loxp}$* LysM+ mice or LysM+ mice (n = 4; Mann-Whitney test). **(E)** ADORA2B protein levels by western blot analysis. **(F)** Quantification of **(E)** (n = 3; two-tailed unpaired t-test). Values are mean ± SEM. Each dot represents 1 mouse.
